# Changes in isokinetic trunk muscle strength and endurance after two different restoration programs in people with chronic low back pain: A longitudinal retrospective study

**DOI:** 10.1016/j.heliyon.2024.e34914

**Published:** 2024-07-20

**Authors:** Marvin Coleman, Jonathan Linières, Camille Thery, Adrien Gautier, Camille Daste, François Rannou, Christelle Nguyen, Marie-Martine Lefèvre-Colau, Alexandra Rören

**Affiliations:** a AP-HP, Centre-Université Paris Cité, Service de Rééducation et de Réadaptation de l’Appareil Locomoteur et des Pathologies du Rachis, Hôpital Cochin, 75014, Paris, France; b Université Paris Cité, Institut des Sciences du Sport-Santé de Paris (URP 3625), F-75015, Paris, France; c Université Paris Cité, INSERM, UMR 1153, Centre de Recherche en Épidémiologie et Statistique Sorbonne Paris Cité, 75004, Paris, France; d Université Paris Cité, Faculté de Santé, UFR de Médecine, 75006, Paris, France; e Fédération pour la Recherche sur le Handicap et l’Autonomie, 75013, Paris, France; f Université Paris Cité, INSERM UMR-S 1124, Toxicité Environnementale, Cibles Thérapeutiques, Signalisation Cellulaire et Biomarqueurs, Campus Saint-Germain-des-Prés, 75006, Paris, France

**Keywords:** Functional restoration program, Low back pain, Back muscles, Muscle strength, Isokinetic

## Abstract

**Background:**

Multidisciplinary functional restoration programs (FRPs) aim to improve pain and function in people with chronic low back pain (CLBP). The intensity and content of FRPs varies; the benefits of one program over another are unclear.

**Objective:**

To assess changes in trunk muscle strength and endurance after an intensive (IFRP) (for people on sick leave for >6 months with high levels of fear-avoidance beliefs about physical activity and work) or semi-intensive (SIFRP) (for people working) FRP in people with CLBP.

**Methods:**

Longitudinal retrospective study from March 2016 to December 2019. Setting: rehabilitation department of a tertiary care center. Trunk flexor and extensor muscle strength (60°.s^−1^) and endurance (120°.s^−1^) were measured with the Humac NORM isokinetic dynamometer at pre and post FRP. Change in isokinetic variables (peak torque, total work and flexor/extensor ratio) after each program was assessed with a paired *t*-test (p < 0.05). Pearson's rho and multiple linear regression assessed associations between changes in isokinetic and clinical variables and demographic characteristics.

**Results:**

125 individuals, 63.2 % female, age 43.5 (10.3) years, were included. Mean low back pain intensity was 49.8 (24.9) and 37.2 (25.8)/100 and mean activity limitation (QBPDS) was 38.8 (16.4) and 32.0 (14.6)/100 in the IFRP and SFRP groups, respectively. Trunk extensor peak torque, flexor total work, extensor total work and flexor/extensor peak ratio improved significantly in both FRPs, p < 0.001. The flexor/extensor total work ratio improved in the IFRP group only, p = 0.003. Trunk extensor endurance increased more in the IFRP than the SIFRP group, the absolute pre-post differences for extensor total work [95%CI] N.m were 611.7 [495.2; 728.3] in the IFRP group and 380.0 [300.8; 459.3] in the SIFRP group. No variables were correlated and none predicted improvement in extensor total work in either group.

**Conclusion:**

This study highlights the short-term independence of clinical and trunk muscle strength and endurance changes.

## Highlights

• Trunk isokinetic strength and endurance significantly improved after both programs.
• The intensive program showed greater improvement in trunk muscle endurance.
• Changes in clinical and isokinetic variables were not significantly correlated.
• No demographic or clinical data predicted extensor endurance improvement.

## 1 Introduction

Low back pain (ICD-10-CM Diagnosis Code M54.5) is a worldwide public health problem. Low back pain is a symptom, rather than a disease [1]. The most common form is non-specific low back pain, which includes various contributors (i.e. pathoanatomical conditions, contextual factors, cognitive and affective dimensions of pain, etc.) and excludes specific diseases or anatomic abnormalities [2,3]. The prevalence of non-specific low back pain can reach 85 % in developed countries and is continuing to increase. It is one of the main causes of years of disability for adults worldwide [4]. Chronic low back pain (CLBP) is defined by symptoms lasting longer than 3 months [5]. A systematic review showed that the prevalence of CLBP varies from 4.2 % to 19.6 %. and increases with age [6] The high, financial cost of low back pain mainly results from CLBP.

CLBP is associated with physical and psychosocial deconditioning [4]. Physical deconditioning is characterized by a decrease in trunk muscle strength and endurance, particularly the extensors [7,8], lumbar spine mobility and cardiovascular capacity [9].

Multidisciplinary functional restoration programs (FRPs) aim to improve physical, psychological and social factors associated with CLBP, and to facilitate return to work in people who have not responded to first-line treatments [4,10]. They combine intensive physical training, behavioral therapy and social management, and require active patient participation [10,11]. FRPs vary in intensity (total duration and duration per day), the proportion of each of the 3 major components (physical, psychological and social), the setting (inpatient or outpatient), and their combination with other work-related interventions (e.g., ergonomics) [10,11]. The benefits of one program over another and the characteristics of the people they are designed for are not clearly established [11,12], but moderate-level evidence shows improvements in pain and function in people with CLBP [11].

Pain, function, and trunk muscle strength and endurance are associated in CLBP. A cross-sectional study including 90 people with CLBP showed an association between trunk muscle endurance and pain [13]. A randomized controlled trial including 36 people with CLBP showed that a 12-week program of trunk muscle strengthening and walking significantly decreased activity limitation [14].

Moderate-level evidence shows improvements in muscular strength and endurance after FRPs [11,15]. Two studies assessed trunk muscle strength and endurance after a 5-week FRP in participants with CLBP [16,17], but only one used an isokinetic device to measure the outcomes [16]. Both studies found significant improvements in trunk muscle isometric endurance [17] or isokinetic strength and endurance [16] after the FRP.

The isokinetic dynamometer is considered the gold standard for assessing muscular strength and endurance [18,19]. A systematic review including 5 studies (141 participants with low back pain) reported inter-session intra- and inter-rater reliability of trunk extensor and flexor strength and endurance using an isokinetic dynamometer [20]. The review found good quality evidence of good to excellent reliability of trunk muscle strength and endurance [20]. The correlations between muscle cross-sectional area and peak torque (0.70 < r < 0.85, p < 0.001) and electromyographic (EMG) activity and submaximal isometric torque (r > 0.99, p < 0.0001) showed good construct validity of trunk muscle strength and endurance measured using an isokinetic device [21].

No studies have compared the effects of different FRPs on muscular strength and endurance or assessed the link between muscular and clinical changes in people with CLBP.

The primary objective of this study was to assess changes in trunk isokinetic muscular strength and endurance after 2 different FRPs, intensive (IFRP) and semi-intensive (SIFRP), in people with CLBP. The secondary aim was to assess the association between changes in isokinetic and clinical variables. Our main hypothesis was that trunk extensor endurance would improve more in the IFRP than in the SIFRP group. We also hypothesized that improvements in trunk isokinetic strength and endurance would be correlated with changes in activity limitation, anxiety and depression, and fear-avoidance beliefs.

Assessing the impact of FRPs of different intensities and the link between muscular and clinical variables in people with CLBP would provide valuable information for clinicians and individuals with CLBP regarding the improvements to be expected. Furthermore, such information would help clinicians and researchers to optimize the content of the programs.

## 2 Material and methods

### 2.1 Design

We conducted a non-interventional longitudinal, single-center retrospective study of systematically collected clinical data related to routine practice without any additional diagnostic measures or monitoring, from the Physical and Rehabilitation Medicine (PRM) department of a tertiary care center (Assistance-Publique-Hôpitaux de Paris, Paris, France) from March 2016 to December 2019. We reported our study in accordance with the STROBE [22] and TIDieR [23] checklists (Supplementary material 1 and 2).

### 2.2 Ethics statement

The study was conducted in accordance with the Helsinki Declaration and the rules of good clinical practice. Because the study was retrospective, individuals were not asked to participate at the time of the FRPs. However, all participants had been informed that their personal data, routinely collected as part of administrative and healthcare management processes, could be used for research purposes. Once the decision was made to use the data for research, participants were informed of the study so that they could object to the use of their data. Informed consent was obtained for publishing the participant's image. The study protocol was approved by our institutional review board (CER #00011928, reference 2022-10-12).

Blinding: The principal investigator (MC) who carried out the outcome assessments was not involved as therapist in the FRP. Nevertheless, he was not blinded to the group (IFRP or SIFRP) as allocation was performed according to participants’ baseline clinical characteristics.

### 2.3 Participants

People who had attended an IFRP or SIFRP and had performed the initial and final isokinetic tests were included. The participants had been prescribed the FRP by a (rheumatologist or PRM physician) from the PRM department, who was an expert in the treatment of CLBP (5–35 years of experience). People were referred to the PRM unit by general practitioners or specialists (rheumatologists etc.) for the treatment of disabling CLBP after conventional treatment failure. Low back pain is a symptom, it describes pain felt between the lower ribs and the buttocks [1 Low back pain (who.int)]. The diagnosis of non-specific low back pain is clinical [24]. It involves a process of elimination, beginning with screening for “red flags” [5]. CLBP is defined by symptoms lasting for a long time (more than 3 months) [5]. A thorough and paraclinical exam may identify the specific etiology. At the stage of CLBP, any degenerative lesions are considered stabilized and they do not influence the content of the FRP.

Allocation to the IFRP or SIFRP was determined by a PRM physician. The inclusion criteria for each FRP differed. They were based on the individual's personal and professional profiles. The personal factors included previous treatment history and factors related to the persistence of pain (high levels of pain intensity and disability, catastrophism, high emotional distress, negative recovery expectations), and were measured using specific clinical scores and the patient's attitude and words during the consultation [25]. The professional profile was based on the individual's professional situation and plan.

The IFRP was dedicated to people with CLBP who had been on sick leave for more than 6 months but planned to return to work at the end of the program, with high levels of fear-avoidance beliefs about physical activity and work, and low levels of physical activity. The main objective was return to work [4,10].

The SIFRP was dedicated to people with CLBP, in active employment on the day of the consultation and with low levels of physical activity and some fear-avoidance beliefs about physical activity and work. The main objective of the SIFRP was to decrease activity limitation [10].

#### 2.3.1 Interventions

The content of both FRPs was defined by a steering committee composed of PRM physicians, rheumatologists, physiotherapists, occupational therapists, adapted physical activity teachers (APAT), psychologists and social workers, all experienced in the management of patients with CLBP. The interventions were carried out by an experienced multidisciplinary team, including physiotherapists, occupational therapists, APATs, psychologists and social workers. The principal investigator (MC) did not participate in the interventions. Both FRPs combined physical activity (aerobic training; muscle strengthening, particularly back muscle endurance; stretching and functional movements) and pain management techniques based on cognitive behavioral therapy [1,10,26] and were conducted in groups of 6–8 participants. The intensity and number of repetitions of the physical exercises were determined according to initial scores and increased throughout the programs.

#### 2.3.2 IFRP

The program was performed 7 h per day, 5 days per week for 5 weeks (175 h including 125 h of physical activity). In addition to physical activity, the IFRP focused strongly on pain management techniques and included the intervention of a social worker to help participants to return to work and a dietician to provide nutrition and dietary advice if needed (Supplementary material 3).

#### 2.3.3 SIFRP

This lighter program was performed 5 h per day, 2 days per week for 4 weeks (40 h, including 32 h of physical activity) (Supplementary material 3).

### 2.4 Outcomes

The primary outcome was the change in trunk isokinetic variables [peak torque, total work of trunk flexors and extensors, and flexor/extensor (F/E) ratio] from the initial (before beginning the program) to the final (end of the program) assessments. Peak torque indicates the highest muscle force output through the range of motion and reflects muscle strength. Total work indicates the total amount of work accomplished during the set of repetitions and reflects muscle endurance. The F/E ratio indicates the strength balance between agonists and antagonists: a F/E ratio <1 indicates stronger extensors than flexors [27]. The isokinetic data were extracted from the routine clinical data collection.

**Fig. 1:**
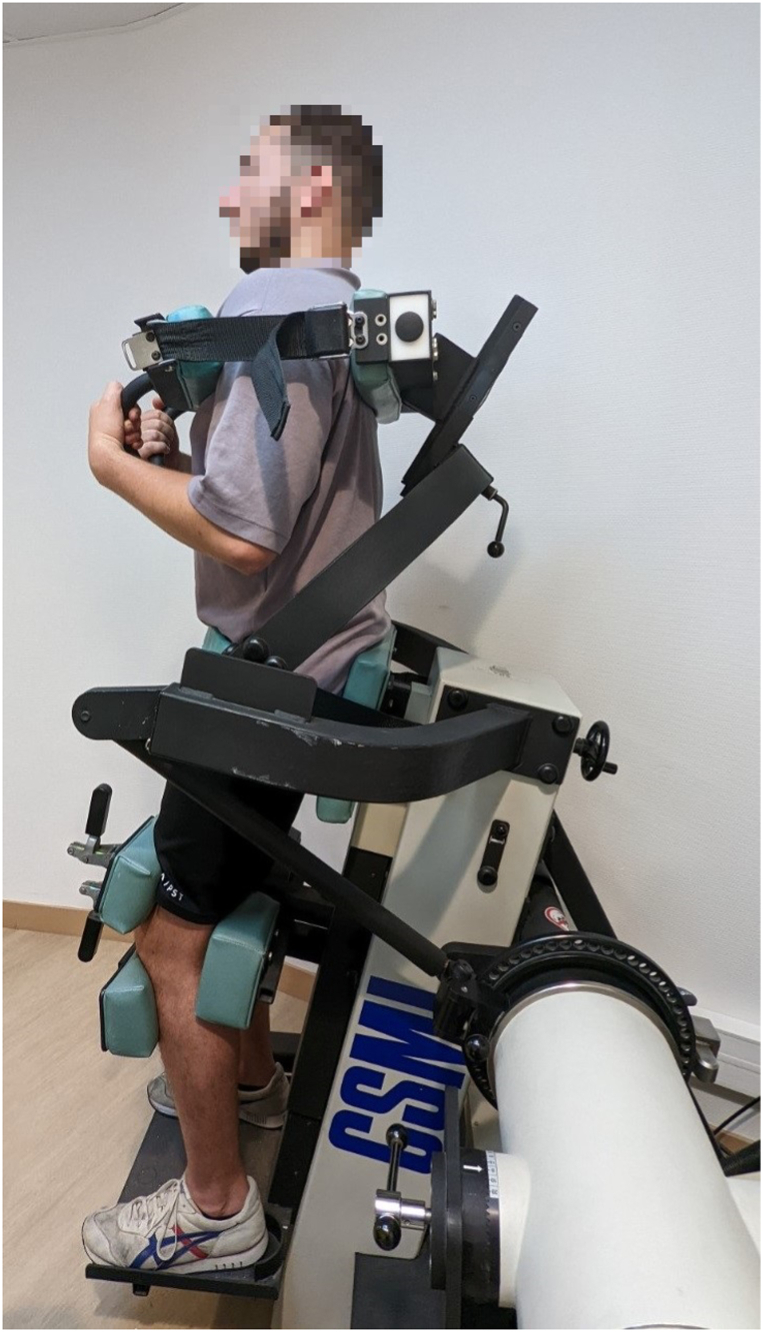
Participant set-up for isokinetic tests.

Trunk muscle strength and endurance tests were performed by one of 2 APATs (JL and AG) with the Humac NORM isokinetic dynamometer (CSMi, Stoughton, MA, Software HUMAC 2009, v.9.7.1) in a dedicated room in the PRM department. Participants first performed a 10-min warm-up with an ergometer at a comfortable speed and resistance that were gradually increased (power from 60 to 90 W). Participants stood on an adjustable horizontal platform so that the dynamometer axis corresponded to the intersection between the horizontal axis L5-S1 and the vertical midaxillary line (Fig. 1). Adjustable pads at the knee, sacrum and upper chest secured the position and minimized hip motion [28]. Trunk range of motion was 75° (70° of trunk flexion and 5° of extension relative to the anatomical reference). Isokinetic concentric strength (60°.s^−1^) and endurance (120°.s^−1^) tests were successively performed. Before the tests, participants had a warm-up and familiarization session, including 2 submaximal trials at 60°.s^−1^ and 5 submaximal trials at 120°.s^−1^. A 60s rest was imposed between the warm-up session and the trials and a 120s rest between the strength and the endurance tests. Participants performed 5 trials at 60°.s^−1^ to assess trunk muscle strength and 20 trials at 120°.s^−1^ to assess trunk muscle endurance [29]. They were told to “go as fast and as hard as possible” to ensure maximal effort throughout the test. Peak torque and total work were expressed in Newton meters (N.m). Data were processed without gravity correction [30]. Isokinetic data measured by the software HUMAC 2009 were anonymized and transferred to an Excel sheet.

The secondary outcomes were change in:

- low back and radicular pain intensity assessed with a self-administrated Numeric Rating Scale (NRS, 0 = no pain to 100 = maximal pain). The NRS (usually rated on 10 points) has high test-retest reliability in people with chronic pain, r > 0.95 [31]. Based on one study, a systematic review reported that the test-retest reliability of the NRS was excellent in adults with low back pain [32], although the results for validity are inconsistent [32]. NRS ratings are highly correlated with VAS ratings in people with chronic pain [31]. A change of 2.5/10 points on the NRS is considered clinically significant in people with CLBP [33]; however, the responsiveness of the NRS appears inconsistent [32].
- activity limitations rated with the self-administered Quebec Back Pain Disability Scale (QBPDS, 0 = no limitation to 100 = maximum limitation) [34,35]. A systematic review including 27 articles found limited-to-moderate evidence of good reliability, validity, and responsiveness of the QBPDS in people with low back pain [36]. The minimal detectable change (the minimal change that falls outside the measurement error) for the QBPDS is 24.6 points in people with CLBP [37].
- levels of anxiety and depression assessed with the Hospital Anxiety and Depression subscales for anxiety (HAD A) and depression (HAD D) (0 = no signs of anxiety or depression to 21 = maximal signs of anxiety or depression). The test-retest reliability of the HAD has not been evaluated. A study showed that the HAD subscales and global scale could detect short-term, significant changes in people with acute low back pain [38]. The HAD demonstrates good criterion-related validity [39]. Correlations between the HAD subscales and other commonly used questionnaires are moderate to very strong, ranging from 0.49 to 0.83 [39]. The minimal clinically important difference (the minimal change in the score that is meaningful for patients) (MCID) of the HAD has not yet been assessed in people with CLBP but is 1.7 points in people with cardiovascular disease [39].
- beliefs about the influence of physical activity and work on low back pain assessed with the subscales of the Fear-Avoidance Beliefs Questionnaire for general physical activity (FABQ-PA) (0 = no fear-avoidance beliefs, 24: maximal fear-avoidance beliefs) and work (FABQ-W) (0 = no fear-avoidance beliefs, 42: maximal fear-avoidance beliefs) [40,41]. The test-retest reliability of the FABQ in people with low back pain is good to excellent [42]. The correlations with another measure of fear-avoidance, the Tampa scale for kinesiophobia (TSK), vary from moderate to strong [42,43]. The minimal detectable changes are 3.7/24 points for the FABQ-PA, and 6.0/42 points for the FABQ-W in Italian adults with CLBP undergoing multidisciplinary rehabilitation [44].
- isometric endurance of the trunk flexors assessed with the (Ito-)Shirado test [45]; and isometric endurance of the trunk extensors assessed with the (Biering-)Sorensen tests [46]. The test-retest reliability of the Shirado test is excellent (ICC>0.95) [47]. The test-retest reliability of the Sorensen test in physically active individuals with low back pain is good to excellent (0.82 <ICC<0.96) [48]. The Shirado test has good criterion-related validity in office workers with nonspecific subacute low back pain [47]. The Sorensen test has good convergent validity (significant relationship with the isokinetic and isometric parameters, r = 0,50 and r = 0.58, respectively, p < 0,001) [49]. A minimal detectable change in the Sorensen test of 24.5s in people with low back pain was estimated from the data of the study of Simmonds et al. (1998) [50]. No minimal detectable or change or MCID data are available for the Shirado test in people with low back pain.

The self-administered questionnaires were completed by participants on the first and last days of the FRP.

Using selected clinical outcomes (low back pain, activity limitation, scores of anxiety and depression and fear and avoidance beliefs regarding work and physical activity) [11], we assessed the association between demographic characteristics (sex and age) and change in clinical and isokinetic variables of interest (flexor and extensor peak torque and total work) [16]. We also tested whether demographic characteristics and changes in clinical scores of interest significantly predicted change in isokinetic extensor endurance in each group. The demographic and clinical data were extracted from medical and paramedical records, anonymized and transferred to an Excel sheet.

### 2.5 Data analysis

#### 2.5.1 Sample size

The study by Caby et al. (2016) showed that trunk extensor endurance (expressed as total work in % of body weight) was 104 (63)% before the FRP and 211 (70)% at the end of the 5 weeks of training [16]. Considering those results with α = 0.05 and 80 % power, the sample size required was 12 participants. The changes reported by Caby et al. [16] are higher than the mean changes that we had found over several years in our unit. According to our data, the expected difference in trunk extensor endurance was 550 (1000) N.m after IFRP and 230 (450) N.m. after SIFRP. With α = 0.05 and 80 % power, the sample size was then 52 and 60 for the IFRP and the SIFRP respectively. Therefore, the sample size of 125 participants (IFRP n = 61 and SIFRP n = 64) should have adequate statistical power.

Systat 12 software was used for data analysis. Quantitative data were expressed as means and % or absolute differences (mean of the absolute value of the differences after excluding missing data) with standard deviations (SD) or 95 % confidence intervals [CI]. Categorical data were expressed as absolute and relative frequencies (n/N [%]). The isokinetic variables of interest were peak torque and total work in Newton meters (N.m) and F/E ratio. We did not statistically compare the baseline characteristics of the IFRP and SIFRP groups since they differed by definition. Given the different sex distribution between the 2 groups and the known relationship between sex and muscle strength and endurance [16,28], baseline isokinetic scores were also expressed by sex.

We used a paired *t*-test to compare initial and final data within each group. We used Pearson's rho to assess correlations between continuous variables of interest: age, changes in clinical (low back pain, activity limitation, anxiety and depression and fear-avoidance beliefs regarding work and physical activity) and isokinetic (flexor and extensor peak torque and total work) variables in the IFRP and SIFRP group, independently. Correlations were defined as weak (0.1< r ≤ 0.3), moderate (0.3< r ≤ 0.5), strong (0.5< r ≤ 0.7) or very strong (r > 0.7) [51]. We used multiple linear regression to test if demographic characteristics (sex and age) and changes in clinical scores of interest (selected if they changed significantly from the initial to final assessment) predicted the change in isokinetic extensor endurance in each group. P < 0.05 was considered statistically significant for all tests.

## 3 Results

### 3.1 Demographic, clinical, and isokinetic data at baseline in both groups

**Fig. 2:**
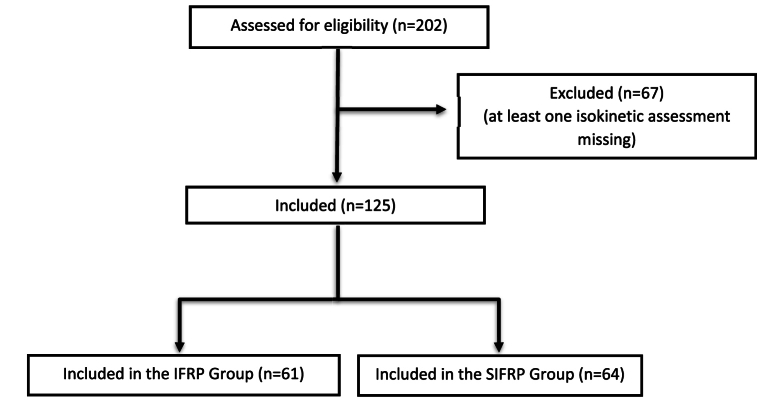
Flow chart.

During the study period, 202 individuals participated in 1 of the 2 FRPs. Seventy-seven were excluded from the study because they did not perform both isokinetic assessment sessions (absent on the day of the final assessment or device unavailable) (Fig. 2).

One hundred and twenty-five individuals, 63.2 % female, age 43.5 (10.3) years, were included in the study. Sixty-one individuals (32 [52.5 %] females, age: 41.7 (9.0) years and BMI: 25.9 (5.1) kg.m-2) were included in the IFRP group and 64 (47 [73.5 %] females, age: 45.3 (11.2) years and BMI: 24.3 (4.5) kg.m-2) in the SIFRP group.

General and clinical participant characteristics and baseline isokinetic values are shown in Table 1a.

**Table 1a:** Participant characteristics at baseline.

	IFRP Group n = 61	SIFRP Group n = 64
General characteristics		
Female n (%)	32 (52.5)	47 (73.5)
Age (years) mean (SD)	41.7 (9.0)	45.3 (11.2)
BMI (kg.m^−2^) mean (SD)	25.9 (5.1)	24.3 (4.5)
Currently working n (%)	3 (5)	56 (84)
Duration of sick leave for LBP (months) mean (SD)	14.2 (9.4)^a^	3 (10.0)^b^
**Clinical characteristics, mean (SD)**		
Low back pain intensity on NRS (0–100)	49.8 (24.9)^c^	37.2 (25.1)^d^
Radicular pain intensity on NRS (0–100)	23.7 (32)^c^	28.7 (30.8)^c^
QBPDS Scale (range 0–100)	38.8 (16.4)^a^	32.0 (14.6)^e^
HAD-D (0–21)	7.8 (4.8)^f^	6.3 (4.3)^g^
HAD-A (0–21)	9.7 (4.2)^f^	10.5 (4.1)^g^
FABQ-W (0–42)	29.6 (8.2)^h^	15.2 (11)^g^
FABQ-PA (0–24)	10.5 (6.2)^h^	10.1 (7.5)^g^
Shirado (s)	84.8 (61.4)^i^	97.1 (51.2)^e^
Sorensen (s)	88.3 (47.5)^d^	94.1 (51.4)^e^
**Main anatomical findings, n (%)**^**j**^		
Previous surgery	11 (18.3)	9 (14.1)
Lumbar spinal stenosis	1 (1.7)	6 (9.4)
Active disc disease	7 (11.7)	14 (21.9)
Disc herniation	8 (13.3)	6 (9.4)
Disc protrusion	10 (16.7)	6 (9.4)
Scheuermann disease	2 (3.3)	3 (4.7)
Degenerative disc disease	31 (51.7)	26 (40.6)
Facet joint osteoarthritis	16 (26.7)	13 (20.3)
Spondylolisthesis	4 (6.7)	7 (10.9)
Lumbar scoliosis	3 (5)	2 (3.1)
Other	12 (20)	13 (20.3)
**Isokinetic variables, mean (SD)**		
Initial flexor peak torque, 60°.s^−1^, (N.m)	186 (5.3)	158.9 (43.2)
Initial extensor peak torque, 60°.s^−1^ (N.m)	211.7 (87.9)	163.8 (62)
Initial F/E peak ratio	0.9 (0.2)	1.0 (0.3)
Initial flexor total work, 120°.s^−1^ (N.m)	2442.5 (1088.5)	2087.4 (806.4)
Initial extensor total work, 120°.s^−1^ (N.m)	2032.0 (1164.3)	1754.4 (898.7)
Initial F/E total work ratio	1.4 (0.6)	1.5 (0.4)

IFRP: intensive functional restoration program; SIFRP: semi-intensive functional restoration program; BMI: Body Mass Index; NRS: numeric scale; QBPDS: Quebec Back Pain Disability Scale; HAD-A: Hospital Anxiety and Depression subscale assessing anxiety; HAD-D: Hospital Anxiety and Depression subscale assessing depression; FABQ-PA: Fear-Avoidance Beliefs Questionnaire related to general physical activity; FABQ-W: Fear-Avoidance Beliefs Questionnaire related to work; Sorensen test: isometric endurance of back extensor muscles; Shirado test: isometric endurance of trunk flexor muscles; N.m: Newton meters. The total number of pathologies exceeds the number of participants because one participant could have more than one etiology (anatomical finding) for LBP: ^a^n = 46; ^b^n = 61 ^c^n = 47, ^d^n = 45, ^e^n = 58 ^f^n = 43, ^g^n = 55, ^h^n = 41, ^i^n = 44, ^j^n = 60.

In both groups, initial flexor and extensor peak torque and total work were lower for females than males (Table 1b).

**Table 1b:** Baseline isokinetic variables by sex.

Isokinetic variables, mean (SD)	IFRP Group n = 61		SIFRP Group n = 64	
	Women n = 32	Men n = 29	Women n = 47	Men n = 17
Flexor peak torque 60°.s^−1^ (N.m)	147.1 (31.4)	228.9 (45.4)	141.4 (28.6)	207.4 (40.1)
Extensor peak torque 60°.s^−1^ (N.m)	161.6 (51.8)	267.1 (86.9)	140.0 (36.5)	229.9 (70.6)
F/E peak ratio 60°.s^−1^	0.9 (0.2)	0.9 (0.3)	1.1 (0.3)	1.0 (0.2)
Flexor total Work 120°.s^−1^ (N.m)	1718.9 (723.3)	3241.0 (837.2)	1743.2 (513.1)	3039.2 (702.3)
Extensor total Work 120°.s^−1^ (N.m)	1406.5 (748.6)	2722.2 (1159.3)	1395.1 (468.4)	2747.7 (1059.3)
F/E total work ratio 120°.s^−1^	1.3 (0.4)	1.4 (0.7)	1.3 (0.4)	1.3 (0.5)

IFRP: intensive functional restoration program; SIFRP: semi-intensive functional restoration program; F/E: flexors/extensors; N.m: Newton meters. Positive changes in peak and total work and negative changes for ratios correspond to improvements.

#### 3.1.1 Between-group differences

The main numerical differences in baseline characteristics between groups were for sex distribution, work activity and duration of sick leave, low back pain and FABQ about work (Table 1A). As compared with the IFRP, the SIFRP group was more predominantly female (+21 %), was more likely to be working at the time of the program (+79 %), had a lower duration of sick leave (absolute difference [95 % CI]: 15.0 [12.0; 17.9] months), lower back pain (absolute difference 23.2 [15.2; 31.3]) and lower FABQ score about work (absolute difference 16.2 [8.6; 16.3]).

The IFRP group had higher initial trunk isokinetic scores than the SIFRP group: the absolute differences [95%CI] were 63.2 [52.6; 73.7] N.m for flexor peak torque, 96.2 [75.2; 117.2] N.m for extensor peak torque, 1204.5 [1017.9; 1391.1] N.m for flexor total work and 1267.7 [1009.9; 1525.6] N.m for extensor total work.

The gender-related major differences between the IFRP and SIFRP groups were for initial flexor peak torque in males and extensor peak torque in both females and males, which were higher in the IFRP group (Table 1B). The absolute differences [95 % CI] were 89.9 [72.6; 101.2] N.m for flexor peak torque in males, 48.0 [30.3; 65.8] N.m for extensor peak torque in females and 119.5 [88.9; 150.1] N.m in males.

### 3.2 Pre-post FRP change in isokinetic variables

#### 3.2.1 IFRP group

Initial and final values are shown in Table 2. Extensor peak torque, F/E peak ratio, flexor and extensor total work improved significantly, p < 0.001, F/E total work ratio improved significantly, p = 0.003. The absolute differences [95 % CI] were 30.1 [23.3; 37.0] N.m for extensor peak torque, 439.8 [338.0; 541.5] N.m and 611.7 [495.2; 728.3] N.m for flexor and extensor total work. The absolute differences [95 % CI] were 0.1 [0.1; 0.1] for F/E peak ratio and 0.3 [0.2; 0.4] for F/E total work ratio. Flexor peak torque did not change significantly.

**Table 2:** Change in isokinetic variables from pre to post FRP.

Isokinetic variables	IFRP Group n = 61			SIFRP Group n = 64		
	Initial	Final	Change (%) p-value	Initial	Final	Change (%) p-value
Flexor peak torque 60°.s^−1^ (N.m)	186 [171.6; 200.4]	190.3 [177; 203.7]	2.3	158.9 [148.1; 169.7]	159.3 [149.5; 169.1]	0.2
			0.046			0.804
Extensor peak torque 60°.s^−1^ (N.m)	211.7 [189.2; 234.2]	235.0 [212.1; 257.9]	**11.0**	163.8 [148.4; 179.3]	186.5 [170.9; 202.2]	**13.8**
			<0.001			<0.001
F/E peak ratio 60°.s^−1^	0.9 [0.9; 1.0]	0.9 [0.8; 0.9]	**−8.1**	1.0 [1.0; 1.1]	0.9 [0.8; 1]	**−13.5**
			<0.001			<0.001
Flexor total Work 120°.s^−1^ (N.m)	2442.5 [2163.7; 2721.3]	2800.4 [2564.6; 3036.3]	**14.7**	2087.4 [1886; 2288.9]	2236.2 [2062.4; 2410.1]	**7.1**
			<0.001			<0.001
Extensor total Work 120°.s^−1^ (N.m)	2032.0 [1733.8; 2330.2]	2565.8 [2280.4; 2851.3]	**26.3**	1754.4 [1529.9; 1978.9]	1985.3 [1762.8; 2207.8]	**13.2**
			<0.001			<0.001
F/E total work ratio 120°.s^−1^	1.4 [1.2; 1.5]	1.2 [1.1; 1.3]	**−13.6**	1.3 [1.2; 1.4]	1.2 [1.1; 1.3]	−16.0
			0.003			0.161

Data are mean [95 % CI]. IFRP: intensive functional restoration program; SIFRP: semi-intensive functional restoration program; F/E: flexor/extensor; N.m: Newton meters. Positive variation for peak and total work and negative variations for ratios correspond to improvements. Change = initial score-final score, positive scores indicate improvement for isokinetic strength and endurance scores (peak torque and total work, respectively) and negative scores indicate improvement for flexor-extensor ratio. **In bold**: significant difference between initial and final scores, p < 0.05.

#### 3.2.2 SIFRP group

Initial and final values are shown in Table 2. Extensor peak torque, F/E peak ratio and flexor and extensor total work improved significantly, p < 0.001. The absolute differences [95 % CI] were 26.7 [20.5; 31.8] N.m for extensor peak torque, 240.9 [177.8; 303.9] N.m, 380.0 [300.8; 459.3] N.m for flexor and extensor total work and 0.2 [0.1; 0.2] for F/E peak ratio. Flexor peak torque and flexor/extensor total work ratio did not change significantly.

#### 3.2.3 Between-group differences

Percentage change in peak torque between the initial and final assessments was higher in the SIFRP than the IFRP group for the extensor peak torque (+13.8 VS +11.0) and F/E peak ratio (−13.5 VS -8.1). Percentage change in flexor (+14.7 VS +7.1) and extensor total work (+26.3 VS +13.2) was higher in the IFRP group (Table 2). The absolute difference in change between the IFRP and the SIFRP group [95 % CI] was 26.9 [21.4; 32.5] N.m for extensor peak torque, 355.0 [256.1; 454.0] N.m for flexor total work and 2089.5 [1894.3; 2.284.7] N.m for extensor total work.

### 3.3 Pre-post FRP change in clinical scores

Low back pain intensity did not change significantly in either the IFRP or the SIFRP group.

#### 3.3.1 IFRP group

Radicular pain (−60.3 %, p = 0.002), QBPDS (−25.7 %, p < 0.001), HAD-D (−30.7 %, p = 0.002), Shirado (+63.8 %, p < 0.001) and Sorensen (+40.3 %, p < 0.001) scores improved significantly (Table 3). Absolute differences [95 % CI] were 19.7 [13.3; 26.2], for radicular pain, −10.3 [6.8; 13.9] for QBPDS, 2.6 [1.8; 3.4] for HAD-D, 63.1 [50.0; 76.3] for Shirado and 46.4 [36.2; 56.5) for Sorensen.

**Table 3:** Changes in clinical scores from pre to post FRP.

Clinical characteristics	IFRP Group n = 61			SIFRP Group n = 64		
	Initial	Final	Change (%) p-value	Initial	Final	Change (%) p-value
Low back pain on NRS (0–100)	49.8 [42.3; 57.3]^a^	37.5 [27.3; 47.6]^b^	−24.7^c^	37.2 [29.8; 44.7]^d^	33.8 [27.1; 40.4]^e^	−9.1^d^
			0.058			0.055
Radicular pain on NRS (0–100)	23.7 [14.3; 33.1]^f^	9.4 [3.3; 15.6]^g^	**−60.3**^h^	28.7 [19.7; 37.8]^f^	20.4 [13; 27.7]^e^	−28.9^f^
			0.002			0.105
QBPDS (0–100)	38.8 (33.9; 43.7]^i^	28.8 (24.7; 32.9]^j^	**−25.7**^k^	32.0 (28.1; 35.8]^l^	26.9 (23.3; 30.5]^m^	**−15.9**^e^
			<0.001			0.007
HAD-D (0–21)	7.8 [6.3; 9.3]^n^	5.4 [4.1; 6.7]^i^	**−30.7**^n^	6.3 [5.2; 7.4]^o^	5.4 [4.4; 6.3]^l^	**−14.3**^n^
			0.002			0.036
HAD-A [0–21)	9.7 [8.4; 11.0]^p^	8.4 [7.1; 9.6]^m^	−13.4^q^	10.5 [9.4; 11.6]^o^	8.9 [7.8; 9.9]^l^	**−15.2**^n^
			0.002			0.007
FABQ-W [0–42)	29.6 [27.1; 32.1]^r^	25.4 [22.1; 28.6]^m^	−14.2^s^	15.2 [12.2; 18.2]^o^	13.9 [10.7; 17.2]^o^	−8.6^t^
			0.064			0.368
FABQ-PA [0–24)	10.5 [8.6; 12.5]^r^	8.1 [5.7; 10.5]^l^	−22.9^q^	10.1 [8.1; 12.1]^o^	5.7 [4.1; 7.2]^o^	**−43.6**^t^
			0.191			<0.001
Shirado [s)	84.8 [66.1; 103.5]^u^	142.7 [123.4; 162]^v^	**63.8**^u^	97.1 [83.6; 110.5]^l^	133.1 [115.9; 150.2]^m^	**37.0**^m^
			<0.001			<0.001
Sorensen [s)	88.3 [74.1; 102.6]^a^	123.9 [107.1; 140.7]^v^	**40.3**^u^	94 [80.5; 107.5]^l^	129.5 [114.6; 144.4]^m^	**37.8**^m^
			<0.001			0.003

Data are mean [95 % CI]. IFRP: intensive functional restoration program; SIFRP: semi-intensive functional restoration program; QBPDS: Quebec Back Pain Disability Scale; HAD-A: Hospital Anxiety and Depression subscale assessing anxiety; HAD-D: Hospital Anxiety and Depression subscale assessing depression; FABQ-PA: Fear-Avoidance Beliefs Questionnaire related to general physical activity; FABQ-W: Fear-Avoidance Beliefs Questionnaire related to work; Negative changes indicate improvement for pain, activity limitation; Positive changes indicate improvement for trunk muscle endurance. ^a^n = 45; ^b^n = 32; ^c^n = 30; ^d^n = 49; ^e^n = 57; ^f^n = 47; ^g^n = 35; ^h^n = 33; ^i^n = 46; ^j^n = 48; ^k^n = 42; ^l^n = 58; ^m^n = 59; ^n^n = 54; ^o^n = 55; ^p^n = 43; ^q^n = 40; ^r^n = 41, ^s^n = 39; ^t^n = 53; ^u^n = 44; ^v^n = 51; **In bold**: significant differences between initial and final scores, p < 0.05.

#### 3.3.2 SIFRP group

QBPDS (−15.9 %, p = 0.007), HAD-D (−14.3 %, p = 0.036), HAD-A (−15.2 %, (p = 0.007), FABQ-PA (−43.6 %, p < 0.001), Shirado (37 %, p = 0.003) and Sorensen (37.8 %, p < 0.001) scores improved significantly (Table 3). Absolute differences [95 % CI] were 10.4 [8.2; 12.5] for QBPDS, 2.6 [2.0; 3.3] for HAD-D, 2.5 [1.8; 3.3] for HAD-A, 5.1 [4.0; 6.2] for FABQ-PA, 45.6 [34.3; 57.0] for Shirado and 47.7 [39.0; 56.4] for Sorensen.

### 3.4 Correlations between isokinetic variables and clinical scores and the linear regression model

#### 3.4.1 IFRP group

All the correlations between age and changes in isokinetic and clinical variables were weak and not significant (r < 0.3, p > 0.05), (Table 4).

**Table 4:**
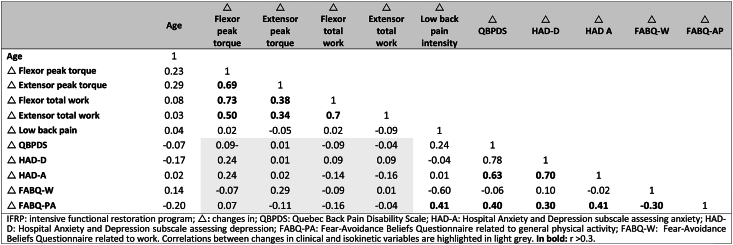
Correlations between demographic characteristics, changes in clinical scores and changes in isokinetic variables in the IFRP group.

The overall regression was not significant, R2 = 0.1; F (6,28) = 0.8; p = 0.54. No demographic characteristics or changes in clinical scores significantly predicted improvement in extensor total work.

#### 3.4.2 SIFRP group

The correlations between changes in flexor peak torque and flexor and extensor total work and change in FABQ-PA score (r = 0.31, r = 0.37 and r = 0.31, respectively) and age and change in FABQ-PA score (r = 0.34) were not significant (p = 0.41, p = 006, p = 0.22 and p = 0.19, respectively) (Table 5).

**Table 5:**
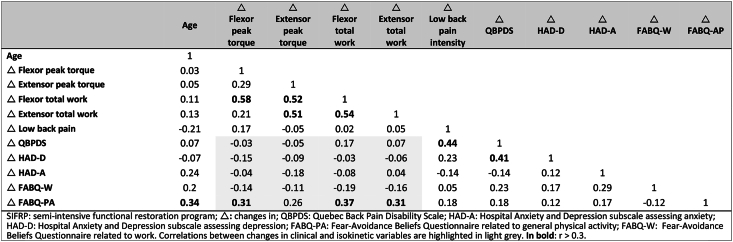
Correlations between demographic characteristics, changes in clinical scores and changes in isokinetic variables in the SIFRP group.

The overall regression was not significant, R2 = 0.1, F (6,41) = 0.7, p = 0.68. No demographic characteristics or changes in clinical scores significantly predicted improvement in extensor total work.

## 4 Discussion

Our study showed significant improvements in trunk isokinetic extensor strength and endurance and flexor endurance after both FRPs. The largest improvement was for extensor endurance in the IFRP group. Changes in isokinetic and clinical variables were not significantly correlated and no changes in clinical scores significantly predicted improvement in extensor total work in any group.

The homogeneity of the assessment and rehabilitation (for each program) is one of the strengths of our study. Nevertheless, the study has several limitations. The groups had different demographic and clinical characteristics at baseline, and allocation was made according to these characteristics. The imbalance in the sex ratio between groups might reduce the external validity of the results. The retrospective and non-controlled, non-randomized design prevents more definitive conclusions about the effect of the interventions. Conclusions are also limited by the very short-term assessments and missing values for clinical tests.

The numeric differences in demographic and baseline clinical characteristics (sex ratio, duration of CLBP-related sick leave, intensity of low back pain, and work-related fear-avoidance beliefs) between the 2 groups reflected the differences in the inclusion criteria in each group. Eight patients in the SIFRP group were on sick leave at the time of the program. This is because the participants’ situations changed between the consultation by the PRM physician and actual beginning of the rehabilitation program.

The higher mean initial flexor and extensor peak torque and flexor total work in the IFRP than the SIFRP group was quite unexpected and underlines the complex relationship between reduced muscle strength and pain, activity, psychological and emotional state in people with CLBP [18]. The differences in initial muscle strength and endurance between groups may be related to between-group differences in the male/female ratio [16]. The higher isokinetic strength and endurance in males compared to females is related to physiology, anthropometric factors: size, weight and weight distribution [52,53], and more experience in producing a maximal effort in males [54].

Trunk extensor peak torque, peak ratio and total work significantly improved in both groups, as previously found [10,16,17]. F/E peak ratio significantly decreased after both FRPs showing that strength and endurance increased more in the extensors than flexors, as previously found [16,18,55]. Because the minimal clinically meaningful difference in the trunk isokinetic variables has not been determined, the clinical relevance of the changes is unclear. In asymptomatic people, muscle endurance shows a dose-response to training volume [56,57]. Four to 6 weeks of high-intensity resistance training (8–12 maximal repetitions, 3 days a week) may be sufficient to increase muscle size and strength [58]. In the current study, we cannot exclude that the improvement in muscle strength was related to non-muscular adaptations, such as a learning effect or a decrease in kinesiophobia and protective behavior [55]. Strength and endurance F/E ratios remained higher than those described in healthy people, indicating the persistence of impairment of trunk extensor strength despite several weeks of FRP [12].

Unlike other studies, the present study assessed 2 different FRPs. In agreement with our main hypothesis, trunk extensor endurance increased more in the IFRP than the SIFRP group. The larger improvement in extensor endurance in the IFRP group favors a more intensive program. Nevertheless, we cannot conclude about the ratio of the intensity of the program/amount of changes in trunk muscle strength and endurance considering the baseline differences in participants’ profiles and different objectives of each FRP.

Low back pain and activity limitation did not improve (the changes were below the MCID or the minimal detectable change) [31,33]. The short-term decrease in pain and activity limitation after FRPs are debated [10,11]. We found significant improvements in fear-avoidance beliefs scores for physical activity in the SIFRP group. We could not state if the change in the anxiety and depression total score was clinically relevant for people with CLBP [59]. Extensor isometric endurance significantly improved in both groups. A longitudinal study of the effect of an IFRP (6 h a day over 5 weeks, 5 days a week) in 144 participants with CLBP found improvements in trunk isokinetic strength, low back pain, daily activities and perceived anxiety-depression at the end of program [16]. Changing work-related fear-avoidance beliefs is a challenge and may require a workplace intervention [60,61].

The current study found that changes in isokinetic variables were not correlated with changes in clinical variables, this result disagreed with our second hypothesis. Associations between changes in isokinetic and clinical variables of interest have never been assessed previously. A controlled study comparing 50 individuals with acute, subacute and CLBP and 50 healthy subjects found no correlation between trunk extensor strength and pain in those with CLBP [62].

The weak correlation between the improvements in strength and endurance and the improvements in pain and function questions the relevance of using trunk muscle strength and endurance to assess improvements in CLBP and raises the issue of using biomedical factors to assess people with CLBP. Nevertheless, core muscle weakness and a lack of coordination are involved in CLBP [63,64]. Trunk muscle strength and endurance contribute to the person's ability to stabilize the lumbar region [65,66]. Changes in muscle strength and endurance may occur before changes in pain and disability. Long-term follow-up might be necessary to assess improvements in clinical symptoms in relation to increased lumbar spine active stability [65].

Isokinetic variables may help clinicians to phenotype individuals with CLBP by specifying their needs and the FRP content and duration and monitoring participants’ progress. FRPs involve high costs for the healthcare system [11,16]. Choosing the most cost-effective program is a critical issue.

## 5 Conclusion

At the end of the FRP programs, trunk isokinetic flexor and extensor strength and extensor endurance improved significantly in both groups. The largest change was for trunk extensor endurance in the participants of the more intensive program. No demographic characteristics or changes in clinical variables were significantly correlated with changes in isokinetic variables in any group underlining the independence of clinical and muscular changes in the short term. Future studies should assess the long-term evolution of muscular strength and endurance and the association with clinical relevant variables (lumbar stability and proprioception, pain and activity limitation)**.**

## Funding

No private or commercial funding was received for the work described in this article.

## Data availability statement

Data will be made available on request.

## CRediT authorship contribution statement

**Marvin Coleman:** Writing – review & editing, Writing – original draft, Formal analysis. **Jonathan Linières APAT:** Writing – review & editing, Formal analysis, Data curation, Conceptualization. **Camille Thery:** Writing – review & editing, Formal analysis, Data curation. **Adrien Gautier APAT:** Writing – review & editing, Formal analysis, Data curation. **Camille Daste:** Writing – review & editing, Methodology, Formal analysis. **François Rannou:** Writing – review & editing, Formal analysis. **Christelle Nguyen:** Writing – review & editing, Formal analysis. **Marie-Martine Lefèvre-Colau:** Writing – review & editing, Writing – original draft, Validation, Supervision, Methodology, Formal analysis, Conceptualization. **Alexandra Rören:** Writing – review & editing, Writing – original draft, Validation, Supervision, Formal analysis, Conceptualization.

## Declaration of competing interest

The authors declare that they have no known competing financial interests or personal relationships that could have appeared to influence the work reported in this paper.

## References

[1] Foster N.E., Anema J.R., Cherkin D., Chou R., Cohen S.P., Gross D.P. (2018). Prevention and treatment of low back pain: evidence, challenges, and promising directions. Lancet.

[2] Saragiotto B.T., Maher C.G., Hancock M.J., Koes B.W. (2017). Subgrouping patients with nonspecific low back pain: hope or hype?. J. Orthop. Sports Phys. Ther..

[3] Dewitte V., De Pauw R., De Meulemeester K., Peersman W., Danneels L., Bouche K. (2018). Clinical classification criteria for nonspecific low back pain: a Delphi-survey of clinical experts. Musculoskelet Sci Pract.

[4] Mayer T.G., Gatchel R.J., Kishino N., Keeley J., Mayer H., Capra P. (1986). A prospective short-term study of chronic low back pain patients utilizing novel objective functional measurement. Pain.

[5] Koes B.W., van Tulder M.W., Thomas S. (2006). Diagnosis and treatment of low back pain. BMJ.

[6] Meucci R.D., Fassa A.G., Faria N.M.X. (2015). Prevalence of chronic low back pain: systematic review. Rev. Saude Publica.

[7] Kerkour K., Meier J. (1994). Évaluation comparative isocinétique des muscles du tronc de sujets sains et de lombalgiques. Ann. Kinésither..

[8] Smeets R.J.E.M., Wade D., Hidding A., Van Leeuwen P.J.C.M., Vlaeyen J.W.S., Knottnerus J.A. (2006). The association of physical deconditioning and chronic low back pain: a hypothesis-oriented systematic review. Disabil. Rehabil..

[9] Verbunt J.A., Seelen H.A., Vlaeyen J.W., van de Heijden G.J., Heuts P.H., Pons K. (2003). Disuse and deconditioning in chronic low back pain: concepts and hypotheses on contributing mechanisms. Eur. J. Pain.

[10] Poiraudeau S., Rannou F., Revel M. (2007). Functional restoration programs for low back pain: a systematic review. Ann. Readapt. Med. Phys.

[11] Kamper S.J., Apeldoorn A.T., Chiarotto A., Smeets R.J.E.M., Ostelo R.W.J.G., Guzman J. (2014). Multidisciplinary biopsychosocial rehabilitation for chronic low back pain. Cochrane Database Syst. Rev..

[12] Mueller S., Stoll J., Mueller J., Mayer F. (2012). Validity of isokinetic trunk measurements with respect to healthy adults, athletes and low back pain patients. Isokinet. Exerc. Sci..

[13] Bozorgmehr A., Zahednejad S., Salehi R., Ansar N.N., Abbasi S., Mohsenifar H. (2018). Relationships between muscular impairments, pain, and disability in patients with chronic nonspecific low back pain: a cross sectional study. J Exerc Rehabil.

[14] Lee J.-S., Kang S.-J. (2016). The effects of strength exercise and walking on lumbar function, pain level, and body composition in chronic back pain patients. J Exerc Rehabil.

[15] Hayden J.A., Ellis J., Ogilvie R., Stewart S.A., Bagg M.K., Stanojevic S. (2021). Some types of exercise are more effective than others in people with chronic low back pain: a network meta-analysis. J. Physiother..

[16] Caby I., Olivier N., Janik F., Vanvelcenaher J., Pelayo P. (2016). A controlled and retrospective study of 144 chronic low back pain patients to evaluate the effectiveness of an intensive functional restoration program in France. Healthcare.

[17] Roche G., Ponthieux A., Parot-Shinkel E., Jousset N., Bontoux L., Dubus V. (2007). Comparison of a functional restoration program with active individual physical therapy for patients with chronic low back pain: a randomized controlled trial. Arch. Phys. Med. Rehabil..

[18] Zouita Ben Moussa A., Zouita S., Ben Salah F., Behm D., Chaouachi A. (2020). Isokinetic trunk strength validity reliability normative data and relation to physical performance and low back pain : review of litterature.

[19] van der Woude D.R., Ruyten T., Bartels B. (2022). Reliability of muscle strength and muscle power assessments using isokinetic dynamometry in neuromuscular diseases: a systematic review. Phys. Ther..

[20] Reyes-Ferrada W., Chirosa-Rios L., Martinez-Garcia D., Rodríguez-Perea Á., Jerez-Mayorga D. (2022). Reliability of trunk strength measurements with an isokinetic dynamometer in non-specific low back pain patients: a systematic review. J. Back Musculoskelet. Rehabil..

[21] Guilhem G., Giroux C., Couturier A., Maffiuletti N.A. (2014). Validity of trunk extensor and flexor torque measurements using isokinetic dynamometry. J. Electromyogr. Kinesiol..

[22] von Elm E., Altman D.G., Egger M., Pocock S.J., Gøtzsche P.C., Vandenbroucke J.P. (2007). The Strengthening the Reporting of Observational Studies in Epidemiology (STROBE) statement: guidelines for reporting observational studies. Bull. World Health Organ..

[23] Hoffmann T.C., Glasziou P.P., Boutron I., Milne R., Perera R., Moher D. (2014). Better reporting of interventions: template for intervention description and replication (TIDieR) checklist and guide. BMJ.

[24] Maher C., Underwood M., Buchbinder R. (2017). Non-specific low back pain. Lancet.

[25] Otero-Ketterer E., Peñacoba-Puente C., Ferreira Pinheiro-Araujo C., Valera-Calero J.A., Ortega-Santiago R. (2022). Biopsychosocial factors for chronicity in individuals with non-specific low back pain: an umbrella review. Int. J. Environ. Res. Publ. Health.

[26] Thomas K., Lee R.Y. (2000). Fatigue of abdominal and paraspinal muscles during sustained loading of the trunk in the coronal plane. Arch. Phys. Med. Rehabil..

[27] Cozette M., Leprêtre P.-M., Doyle C., Weissland T. (2019). Isokinetic strength ratios: conventional methods, current limits and perspectives. Front. Physiol..

[28] García-Vaquero M.P., Barbado D., Juan-Recio C., López-Valenciano A., Vera-Garcia F.J. (2020). Isokinetic trunk flexion-extension protocol to assess trunk muscle strength and endurance: reliability, learning effect, and sex differences. J Sport Health Sci.

[29] Kannus P. (1994). Isokinetic evaluation of muscular performance: implications for muscle testing and rehabilitation. Int. J. Sports Med..

[30] Vanhee J.L., Voisin P h, Vezirian T h, Vanvelcenaher J. (1996). Isokinetic trunk flexors and extensors performance with and without gravity correction. Isokinet. Exerc. Sci..

[31] Hawker G.A., Mian S., Kendzerska T., French M. (2011). Measures of adult pain: visual analog scale for pain (VAS pain), numeric rating scale for pain (NRS pain), McGill pain questionnaire (MPQ), short-form McGill pain questionnaire (SF-MPQ), chronic pain grade scale (CPGS), short form-36 bodily pain scale (SF-36 BPS), and measure of intermittent and constant osteoarthritis pain (ICOAP). Arthritis Care Res..

[32] Chiarotto A., Maxwell L.J., Ostelo R.W., Boers M., Tugwell P., Terwee C.B. (2019). Measurement properties of visual analogue scale, numeric rating scale, and pain severity subscale of the brief pain inventory in patients with low back pain: a systematic review. J. Pain.

[33] Ostelo R.W.J.G., de Vet H.C.W. (2005). Clinically important outcomes in low back pain. Best Pract. Res. Clin. Rheumatol..

[34] Kopec J.A., Esdaile J.M., Abrahamowicz M., Abenhaim L., Wood-Dauphinee S., Lamping D.L. (1995). The Quebec back pain disability scale. Measurement properties. Spine.

[35] Yvanes-Thomas M., Calmels P., Béthoux F., Richard A., Nayme P., Payre D. (2002). Validity of the French-language version of the Quebec back pain disability scale in low back pain patients in France. Joint Bone Spine.

[36] Speksnijder C.M., Koppenaal T., Knottnerus J.A., Spigt M., Staal J.B., Terwee C.B. (2016). Measurement properties of the Quebec back pain disability scale in patients with nonspecific low back pain: systematic review. Phys. Ther..

[37] van der Roer N., Ostelo R.W.J.G., Bekkering G.E., van Tulder M.W., de Vet H.C.W. (2006). Minimal clinically important change for pain intensity, functional status, and general health status in patients with nonspecific low back pain. Spine.

[38] Turk D.C., Dworkin R.H., Trudeau J.J., Benson C., Biondi D.M., Katz N.P. (2015). Validation of the hospital anxiety and depression scale in patients with acute low back pain. J. Pain.

[39] Lemay K.R., Tulloch H.E., Pipe A.L., Reed J.L. (2019). Establishing the minimal clinically important difference for the hospital anxiety and depression scale in patients with cardiovascular disease. J Cardiopulm Rehabil Prev.

[40] Waddell G., Newton M., Henderson I., Somerville D., Main C.J. (1993). A Fear-Avoidance Beliefs Questionnaire (FABQ) and the role of fear-avoidance beliefs in chronic low back pain and disability. Pain.

[41] Chaory K., Fayad F., Rannou F., Lefèvre-Colau M.-M., Fermanian J., Revel M. (2004).

[42] Williamson E. (2006). Fear avoidance beliefs questionnaire (FABQ). Aust. J. Physiother..

[43] Crombez G., Vlaeyen J.W., Heuts P.H., Lysens R. (1999). Pain-related fear is more disabling than pain itself: evidence on the role of pain-related fear in chronic back pain disability. Pain.

[44] Monticone M., Frigau L., Vernon H., Rocca B., Giordano A., Simone Vullo S. (2020). Reliability, responsiveness and minimal clinically important difference of the two Fear Avoidance and Beliefs Questionnaire scales in Italian subjects with chronic low back pain undergoing multidisciplinary rehabilitation. Eur. J. Phys. Rehabil. Med..

[45] Ito T., Shirado O., Suzuki H., Takahashi M., Kaneda K., Strax T.E. (1996). Lumbar trunk muscle endurance testing: an inexpensive alternative to a machine for evaluation. Arch. Phys. Med. Rehabil..

[46] Biering-Sørensen F. (1984). Physical measurements as risk indicators for low-back trouble over a one-year period. Spine.

[47] del Pozo-Cruz B., Mocholi M.H., del Pozo-Cruz J., Parraca J.A., Adsuar J.C., Gusi N. (2014). Reliability and validity of lumbar and abdominal trunk muscle endurance tests in office workers with nonspecific subacute low back pain. J. Back Musculoskelet. Rehabil..

[48] Moreau C.E., Green B.N., Johnson C.D., Moreau S.R. (2001). Isometric back extension endurance tests: a review of the literature. J. Manip. Physiol. Ther..

[49] Ghroubi S., Jribi S., Jdidi J., Yahia A., Elleuch W., Chaaben M. (2015). Study of the validity and reproducibility of the Biering-Sorensen test in chronic low back pain. Annals of Physical and Rehabilitation Medicine.

[50] Simmonds M.J., Olson S.L., Jones S., Hussein T., Lee C.E., Novy D. (1998). Psychometric characteristics and clinical usefulness of physical performance tests in patients with low back pain. Spine.

[51] Cohen J. (1988). Set correlation and contingency tables. Appl. Psychol. Meas..

[52] Danneskiold-Samsøe B., Bartels E.M., Bülow P.M., Lund H., Stockmarr A., Holm C.C. (2009). Isokinetic and isometric muscle strength in a healthy population with special reference to age and gender. Acta Physiol..

[53] Philippaerts R.M., Vaeyens R., Janssens M., Van Renterghem B., Matthys D., Craen R. (2006). The relationship between peak height velocity and physical performance in youth soccer players. J. Sports Sci..

[54] Keller A., Hellesnes J., Brox J.I. (2001). Reliability of the isokinetic trunk extensor test, Biering-Sørensen test, and Astrand bicycle test: assessment of intraclass correlation coefficient and critical difference in patients with chronic low back pain and healthy individuals. Spine.

[55] Vlaeyen J.W.S., Linton S.J. (2000). Fear-avoidance and its consequences in chronic musculoskeletal pain: a state of the art. Pain.

[56] Radaelli R., Fleck S.J., Leite T., Leite R.D., Pinto R.S., Fernandes L. (2015). Dose-response of 1, 3, and 5 sets of resistance exercise on strength, local muscular endurance, and hypertrophy. J. Strength Condit Res..

[57] American College of Sports Medicine (2009). American College of Sports Medicine position stand. Progression models in resistance training for healthy adults. Med. Sci. Sports Exerc..

[58] Abe T., DeHoyos D.V., Pollock M.L., Garzarella L. (2000). Time course for strength and muscle thickness changes following upper and lower body resistance training in men and women. Eur. J. Appl. Physiol..

[59] Bjelland I., Dahl A.A., Haug T.T., Neckelmann D. (2002). The validity of the Hospital Anxiety and Depression Scale. An updated literature review. J. Psychosom. Res..

[60] Coole C., Drummond A., Watson P.J. (2013). Individual work support for employed patients with low back pain: a randomized controlled pilot trial. Clin. Rehabil..

[61] Gatchel R.J., Neblett R., Kishino N., Ray C.T. (2016). Fear-avoidance beliefs and chronic pain. J. Orthop. Sports Phys. Ther..

[62] Gabr W., Eweda R. (2019). Isokinetic strength of trunk flexors and extensors muscles in adult men with and without nonspecific back pain: a comparative study. Journal of Behavioural and Brain Science.

[63] Hammill R.R., Beazell J.R., Hart J.M. (2008). Neuromuscular consequences of low back pain and core dysfunction. Clin. Sports Med..

[64] Tong M.H., Mousavi S.J., Kiers H., Ferreira P., Refshauge K., van Dieën J. (2017). Is there a relationship between lumbar proprioception and low back pain? A systematic review with meta-analysis. Arch. Phys. Med. Rehabil..

[65] Inani S.B., Selkar S.P. (2013). Effect of core stabilization exercises versus conventional exercises on pain and functional status in patients with non-specific low back pain: a randomized clinical trial. J. Back Musculoskelet. Rehabil..

[66] Gordon R., Bloxham S. (2016). A systematic review of the effects of exercise and physical activity on non-specific chronic low back pain. Healthcare.

