# Supplementary material for: Changes in isokinetic trunk muscle strength and endurance after two different restoration programs in people with chronic low back pain: A longitudinal retrospective study
Source: Heliyon. 2024 Jul 20;10(15):e34914. doi: 10.1016/j.heliyon.2024.e34914 (PMC11320202; doi:10.1016/j.heliyon.2024.e34914)
Supplement: Multimedia component 3 [file mmc3.docx]

**Schedule for the intensive functional program (IFRP)**

| **Daily activities** | **5 week program, 7 hours /day, 5 days /week.** |
| --- | --- |
| Warm-up | Joint and muscle warm up (20min), the session is led by a physiotherapist or an APAT |
| Dynamic Stretching | Low back and lower body (45min), the session is led by a physiotherapist or an APAT |
| Muscle strengthening | Core, upper and lower extremities (45min), the session is led by a physiotherapist or an APAT |
| Aerobic training | Cycle ergometer with power progression or running (30min to 45min), the session is led by an APAT |
| Occupational therapy | Professional situation and daily activities (45min), the session is led by an occupational therapist |
| Aerobic training | Cycle ergometer with power progression (30min to 45min), the session is led by an APAT |
| Varied activities | Muscle strengthening, proprioception, dancing, sham boxing or therapeutic education (60min), the session is led by a physiotherapist or an APAT. |
| Hydrotherapy | Aerobic training, stretching, full body strengthening (45min), the session is led by an APAT. |
| Specific interventions |  |
| Medical consultation | There is a medical consultation (with a rheumatologist or a PRM doctor) at the beginning and the end of the program  Patients can meet a physician from the unit during the program if necessary |
| Physical tests | Physical tests (static and isokinetic trunk muscle measurements) and functional tests (ability to lift loads) are assessed at the beginning and the end of the program. |
| Dietitian | The dietitian presents the main principles of a balanced diet (30min).  A dietician meets patients upon request |
| Social worker | The social worker presents his/her role and the help he/she can provide (45min). The social worker provides individual support on request. |
| Psychologist | The psychologist leads two group workshops:  - Work on stress and pain management (60min) (fears and beliefs, the link between thoughts, emotions, and behaviors, the notion of coping, stress moderators etc.).  - Relaxation: Jacobsen techniques and sophrology, (60min). The aim is for the patient to learn to apply these techniques themselves.  The psychologist can provide a more individual follow-up on request. |
| Multidisciplinary meeting | Each week, the multidisciplinary team meets with the patient to discuss their situation and progress (group time: 15min, individual time: 10min). |

**Schedule for the semi-intensive functional program (SIFRP)**

| **Daily activities** | **4 weeks, 2 half-days / week** |
| --- | --- |
| Hydrotherapy | Aerobic, stretching, full body strengthening (45min), the session is led by a physiotherapist or an APAT |
| Occupational therapy | Professional situation and daily activities (45min), the session is led by an occupational therapist |
| Aerobic training | Cycle ergometer with power progression (30min), the session is led by an APAT |
| Dynamic Stretching | Low back and lower body (15min), the session is led by a physiotherapist or an APAT |
| Muscle strengthening | Core, upper and lower extremities (30min), the session is led by a physiotherapist or an APAT |
| Proprioception | Trunk and lower body (30min), the session is led by a physiotherapist or an APAT |
| Specific interventions |  |
| Medical consultation | There is a medical consultation (with a rheumatologist or a PRM doctor) at the beginning and the end of the program  Patients can meet a physician of the unit during the program if necessary |
| Physical tests | Physical tests (static and isokinetic trunk muscle measurements) and functional tests (ability to lift loads) are assessed at the beginning and the end of the program. |
| Dietitian | A dietitian meets patients upon request |
| Social worker | The social worker presents his/her role and the help he/she can provide on the first day of the program. The social worker provides more individual support on request. |
| Psychologist | The psychologist leads two group workshops:  - Work on stress and pain management (45min) (fears and beliefs, the link between thoughts, emotions, and behaviors, the notion of coping, stress moderators etc.).  - Relaxation: (self-)relaxation training using breathing techniques .  The psychologist can provide a more individual follow-up on request. |
