# Supplementary material for: Changes in isokinetic trunk muscle strength and endurance after two different restoration programs in people with chronic low back pain: A longitudinal retrospective study
Source: Heliyon. 2024 Jul 20;10(15):e34914. doi: 10.1016/j.heliyon.2024.e34914 (PMC11320202; doi:10.1016/j.heliyon.2024.e34914)
Supplement: Multimedia component 4 [file mmc4.doc]

| **N° enregistrement du dossier** | reçu par CERAPHP.5 le : |
| --- | --- |
|  |  |
| **Titre de l’étude** | |

**Variation des paramètres spatio-temporels de la stabilité posturale et de la marche chez les personnes lombalgiques chroniques après programme de rééducation supervisé (MarchStaLomb)**

| **Investigateur principal** | |
| --- | --- |
| Nom Prénom :  Structure : | ROREN Alexandra  Hôpital Cochin, AP-HP/département des ciences de la rééducation et de la Réadaptation, Université paris Cité |
| Fonction : | Masseur-kinésithérapeute, Maître de conférences |
| Service / UF : | Service de Rééducation et Réadaptation de l'Appareil Locomoteur et des Pathologies du Rachis/ CRESS-UMR1153, équipe ECaMO |
| Pôle : | DMU Appareil Locomoteur |
| Numéro de téléphone : | 01 58 41 13 71 / 06 22 62 00 50 |
| Email : | alexandra.roren@aphp.fr |
| Login APH : | 3047372 |

| **Co-Investigateur** (*facultatif)* | |
| --- | --- |
| Nom Prénom :  Structure : | Lefèvre-Colau Marie-Martine  Hôpital Cochin, AP-HP |
| Fonction : | MCU-PH |
| Service / UF : | Service de Rééducation et Réadaptation de l'Appareil Locomoteur et des Pathologies du Rachis |
| Pôle : | DMU locomoteur |
| Numéro de téléphone : | 0158412542 |
| Email : | marie-martine.lefevre-colau@aphp.fr |
| Login APH : | 542895 |

*Médecin ou Pharmacien thèsé ou non qui aura aussi accès aux données****.*** *Par exemple un interne*

**Merci de contacter** [demandes.cdw@egp.aphp.fr](mailto:demandes.cdw@egp.aphp.fr) **pour vérifier que les patients concernés par votre recherche ne se sont pas opposés à la réutilisation de leurs données pour la recherche**

**Les investigateurs s'engagent à faire un résumé grand public des résultats de leur recherche à destination des patients qui sera diffusé via un portail de transparence.**

Cette recherche n'est pas une recherche impliquant la personne humaine: OUI / **NON**

Prévoit-elle l’utilisation d’échantillons biologiques humains : OUI / **NON**

Déclaration au DPO (délégué à la protection des données) de l’institution : **OUI** / NON

| Date de la demande : | 25/08/2022 |
| --- | --- |
| Signature obligatoire de l’investigateur principal. | 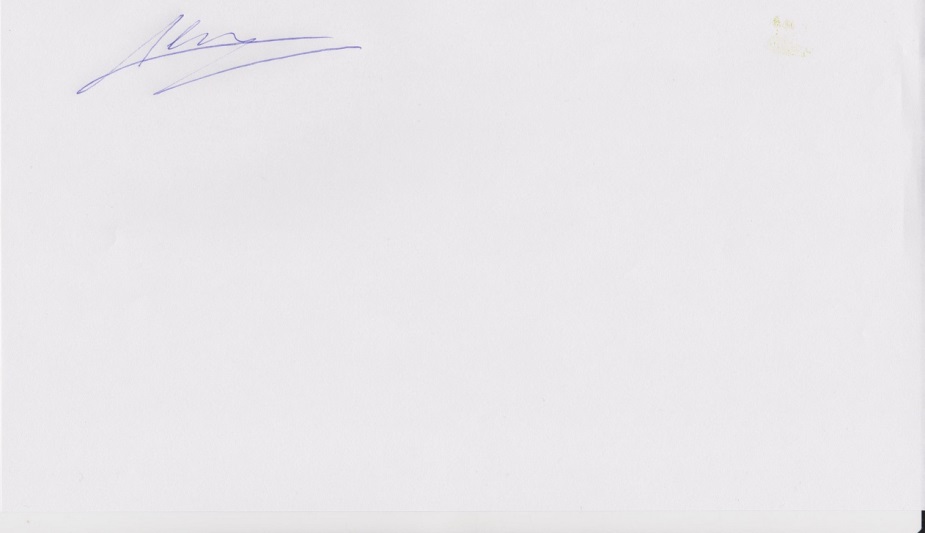 |

| **Accord du chef de service de l’investigateur principal** | | | |
| --- | --- | --- | --- |
|  | Nom Prénom | Rannou François |  |
|  | Service | Service de Rééducation et Réadaptation de l'Appareil Locomoteur et des Pathologies du Rachis |  |
|  | Signature obligatoire | 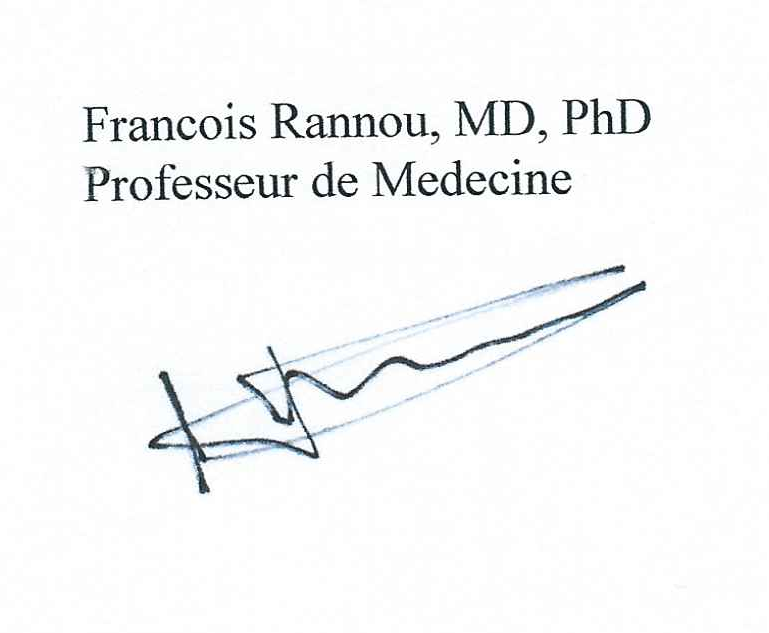 |  |

| **Partenaires associés** | | | |
| --- | --- | --- | --- |
| Nom Prénom : |  | Service / UF : |  |
| Email : |  | Pôle : |  |
| Nom Prénom : |  | Service / UF : |  |
| Email : |  | Pôle : |  |
| Nom Prénom : |  | Service / UF : |  |
| Email : |  | Pôle : |  |
| Nom Prénom : |  | Service / UF : |  |
| Email : |  | Pôle : |  |
| Nom Prénom : |  | Service / UF : |  |
| Email : |  | Pôle : |  |
| Nom Prénom : |  | Service / UF : |  |
| Email : |  | Pôle : |  |
| Nom Prénom : |  | Service / UF : |  |
| Email : |  | Pôle : |  |

*Les Partenaires associés n’auront pas d’accès aux données mais* *ils**seront informés par email de la décision de CERAPHP.5 avec l’envoi de ce document.*

| **Résumé de l’étude**  *Une page* ***minimum et maximum*** |
| --- |

**Contexte de l’étude** :

La lombalgie est définie par une perception douloureuse localisée entre la cage thoracique et le pli fessier inférieur. La lombalgie non spécifique, dont sont exclues les causes tumorales, infectieuses, inflammatoires ou traumatiques, est très fréquente, sa prévalence sur la vie entière varie entre 70 et 85% selon les études. La lombalgie non-spécifique est un problème mondial de santé publique, c’est la première cause d’années vécues en situation de handicap dans le monde et la sixième en matière de réduction de l’espérance de vie en bonne santé. Le pronostic de la lombalgie aiguë est excellent, la disparition des symptômes est obtenue en quelques semaines pour 90% des patients. L’évolution vers la chronicité survient dans 6 à 8% des cas, la lombalgie chronique est à l’origine de coûts médicaux, financiers et humains majeurs.

La lombalgie chronique est associée à des limitations des activités simples de la vie quotidienne. Les personnes lombalgiques chroniques sont caractérisées par une altération de la stabilité posturale et de la marche. (Ruhe et al., 2011 ; Koch et Hänsel, 2018). L’altération de la stabilité posturale est caractérisée par une augmentation de la surface et vitesse de dépacement du centre de pression (point d’application de la résultante des forces de réaction verticale) en position debout statique (Ruhe et al., 2011). Les personnes lombalgiques chroniques marchent significativement moins rapidement que les personnes asymptomatiques appariées en âge et en sexe (Smith et al., 2022). On observe égalament une altération de la dissociation des ceintures (diminution du mouvement coordonné d’opposition des ceintures scapulaires et pelviennes) chez les personnes lombalgiques par rapport à des personnes asymptomatiques (Koch et Hänsel, 2018).

Le Service de Rééducation et Réadaptation de l'Appareil Locomoteur et des Pathologies du Rachis de l’hôpital Cochin possède depuis quelques années un nouveau dispositif de mesure (dispositif Abilycare, labelisé matériel médical) comprenant une plateforme de stabilométrie permettant d’évaluer les paramètres spatio-temporels de la stabilité posturale et des centrales inertielles permettant de mesurer les paramètres spatio-temporels de la marche.

La rééducation basée sur des exercices physiques est le traitement qui a le mieux fait preuve de son efficacité chez les personnes lombalgiques chroniques or nous ne connaissons pas l’effet des programmes de rééducation basés sur les exercices physiques sur les paramètres spatio-temporels de la stabilité posturale et de la marche.

Une évaluation de ces paramètres est réalisée systématiquement dans le cadre du soin, chez les personnes hospitalisées dans le service pour rééducation de la lombalgie chronique, dans le cadre de différents programmes de rééducation. L’ensemble des évaluations initiales (bilan clinique, questionnaires auto-administrés, tests physiques) permet de détecter les déficiences spécifiques, les limitations d’activité et restrictions de participation et de personnaliser le contenu du programme de rééducation supervisé. Les évaluations finales permettent de mesurer les variations après programme de rééducation,

A ce jour, les programmes de rééducation proposés dans notre service, n’incluent pas d’exercices spécifiques d’équilibre et de marche. Jusqu’à présent, les paramètres spatio-temporels de la stabilité posturale et de la marche avant/après programme n’ont été analysées qu’à l’échelle individuelle. Nous n’avons jamais mesuré les variations de ces paramètres dans nos populations de patients lombalgiques.

**Objectif principal de l’étude :**

Evaluer les variations des paramètres spatio-temporels de la stabilité posturale et de la marche chez les patients ayant suivi un programme de rééducation pour lombalgie chronique dans notre service.

**Durée de l’étude :**

Etude rétrospective sur données recueillies dans le cadre des soins entre 2016 et 2022

Retrospective study of routinely collected data between 2016 and 2022

**Critères d’inclusion / non inclusion :**

Critères d’inclusion :

- Age ≥ 18 ans,

- Lombalgie chronique (durée ≥ 3 mois),

- Pris en charge pour lomblagie chronique en hôpital conventionnel ou de jour dans le service de rééducation de l’hôpital Cochin,

Critères de non inclusion :

- Pathologies vestibulaires ou neurologiques,

- Douleur radiculaire prédominante sur la douleur lombaire,

- Grossesse en cours,

- Troubles de la compréhension,

- Maitrise insuffisante de la langue française.

**Type d’étude :** Etude, retrospective, observationnelle, monocentrique

**Méthodologie** *(5 lignes minimum)***:**

Aucun acte ou procédure ne sont ajoutés par la recherche Une lettre de non opposition à l’utilisation de ses données cliniques pour la recherche et expliquant les conditions d’utilisation de ses données personnelles sera adressée à chaque patient.

**Variables extraites pertinentes pour ce projet** :

Critère principal de jugement : variation avant et après programme de rééducation supervisée dans le service de rééducation, des paramètres saptio-temporels de la stabilité posturale (longueur et surface de déplacement du centre de pression) et de la marche (vitesse de marche, temps de double appui, vitesse angulaire de rotation du bassin)

Critères secondaires :

Variation avant et après programme de rééducation supervisée dans le service de rééducation des paramètres suivants :

- Force et endurance isocinétique (en N.m) des muscles fléchisseurs et extenseurs du rachis

Isokinetic strength and endurance of trunk flexors and extensors(in N.m)

- Intensité de la douleur, (EVA, 0-100, 0 absence de douleurs,100 douleur maximale imaginable)
- Limitation d’activité spécifique à la personne lomblagique (questionnaire auto-administré Quebec, 0= absence de limitation, 100 : limitations maximales)
- Peurs et croyances eronnées relatives à l’activité physique et au travail (questionnaire auto-administré FABQ), sous-score activité physique (FABQ-AP, 0 : absence de peurs et croyances erronnées, 24: peurs et croyances erronnées maximales) et sous-score travail (FABQ-travail, 0 : absence de peurs et croyances erronnées,42: peurs et croyances erronnées maximales).
- Anxiété et de dépression (questionnaire auto-adminiqtré HAD), sous-score anxiété (HAD-A, 0 : absence de signe d’anxiété, 21 signes maximaux d’anxiété) et sous-score dépression (HAD-D, 0 : absence de signe de dépression, 21 signes maximaux de dépression).
- **Circuit des données :**

.Les données sont issues du dossier médical numérique Orbis, L’accès à ce serveur sécurisé est restreint aux personnes habilitées, il est sécurisé par un n°identifiant et un mot de passe, et tracé. Les données des patients seront anonymisées par attribution d’un numéro d’anonymisation, les résultats (tableur Excel et fichier de traitement statistique) seront stockées dans le service, **dans une pièce fermée à clef, sur un poste informatique dont l’accès est restreint et sécurisé par un n°identifiant et un mot de passe.**

Les données ne seront pas identifiées par date, les interventions (les programmes de rééducation pour lombalgie chronique) que nous souhaitons analyser par une méthode avant/après, seront identifiées par un numéro de programme et au moyen du délai par rapport à l’évaluation intiale.

**Résultats attendus :** *(si applicable)*

Nous prévoyons d’inclure 200 patients dans cette étude rétrospective. Nous anticipons une amélioration moyenne des paramètres spatio-temporels de la stabilité posturale et de la marche de faible ampleur, après programme de rééducation. Nous faisons l’hypothèse que les paramètres mesurés après programme de rééducation chez la personne lombalgique chronique, demeurent altérés par rapport à ceux décrits dans la littérature chez la personne asymptomatique.

**Références clés** *(1 à 3)***:**

Ruhe A, Fejer R, Walker B. Center of pressure excursion as a measure of balance performance in patients with non-specific low back pain compared to healthy controls: a systematic review of the literature. Eur Spine J. 2011 Mar;20(3):358-68. doi: 10.1007/s00586-010-1543-2.

Koch C, Hänsel F. Chronic Non-specific Low Back Pain and Motor Control During Gait. Front Psychol. 2018;9:2236. doi: 10.3389/fpsyg.2019.00586.

Smith JA, Stabbert H, Bagwell JJ, Teng HL, Wade V, Lee SP. Do people with low back pain walk differently? A systematic review and meta-analysis. J Sport Health Sci. 2022. doi: 10.1016/j.jshs.2022.02.001.

| **Informations complémentaires relatives à l’étude**  *Une page* ***minimum et maximum*** |
| --- |

**Finalité de l’étude :**

Cette étude permettra d’avoir des éléments de réponse sur l’impact à très court terme des programmes de rééducation sur les paramètres spatio-temporels de stabilité posturale et de marche chez la personne lombalgique chronique. A la suite de cette étude, nous pourrons évaluer les liens existants entre paramètres spatio-temporels de la stabilité posturale et de la marche et douleur et fonction et envisager une étude contrôlée randomisée visant à évaluer l’efficacité d’une prise en charge rééducative contenant des exercices spécifiques d’équilibre et de marche sur la douleur et la fonction, des personnes lombalgiques chroniques.

**Nom du responsable du traitement :**

AP-HP représentée par l’hôpital Cochin

**Catégories de données traitées :**

Données cliniques non sensibles quantitatives et qualitatives recueillies dans le cadre du soin et issues du dossier médical.

**Durées de conservation des données utilisées :** au maximum : 2 ans après la dernière publication.

**Destinataire(s) des données traitées (ex : sous-traitants, partenaires accédant aux données, etc.) :**

L’ensemble des données sera traité en interne par l’investigateur principal au sein du service de rééducation et réadaptation de l'appareil locomoteur et des pathologies du rachis de l’hôpital Cochin.

Cette page concerne uniquement les demandes d’accès à l’entrepôt de données clinique du GH UPO. Toutes les informations sont sur : <http://cdw.egp.aphp.fr/wiki>

Veuillez contacter demandes.cdw@egp.aphp.fr pour la validation technique de la demande

| **Descriptif de la requête de sélection des cas** | |
| --- | --- |
| **Critère d'inclusion** | *Exemple avec des codes CIM10 :*  *Infartus du myocarde : I21**  ***ET***  *Embolie Pulmonaire :I26** |
| **Période** | *Exemple: 2000 à 2012* |
| **Nombre de cas** | *Exemple : 85* |
| **Niveau d’accès aux données individuelles** | |

**1) Création d’une liste de patients (sans données cliniques) :**

Je souhaite accéder uniquement à la liste des NIPet NDA des patients.

- **Liste_niveau_3 :** Liste des NIP et NDA décryptés (les vrais NIP et NDA sont affichés, donc identification des patients)

**OU**

**2) Création d’une base de donnée sécurisée PROJET_CDW**

Je souhaite accéder à la liste des NIP et NDA des patients et je souhaite accéder à une base de donnée sécurisée qui ne sera accessible qu’aux personnes mentionnés sur la première page (investigateur principal et co-investigateur) dans l’enceinte du GH UPO.

- **PROJET_CDW_niveau_2**: NIP et NDA cryptés (Impossible de revenir à l’identité des patients. Il reste possible de chainer les données cliniques d’un même patient)
- **PROJET_CDW_niveau_3**: NIP et NDA décryptés (les vrais NIP et NDA sont affichés, donc identification des patients)

| **Niveau d’accès demandé** | **Exemple : PROJET_CDW_niveau_3** |
| --- | --- |
| **Justification du niveau d’accès**  **(obligatoire pour niveaux 3)** | *Exemple : retour au dossier papier nécessaire. Merci de détailler pourquoi.* |
| **Durée de mise à disposition du projet** | *Exemple 2 ans* |

| Validation technique de la demande  par le service informatique et santé publique du GH HUPO  Démarche qualité permettant de vérifier l’adéquation entre le résumé du projet et la nature de la requête envisagée et des variables d’intérêts demandées | |
| --- | --- |
| Date |  |
| Nom Prénom |  |
| Signature d’un médecin du service de santé publique et informatique médicale du GH UPO |  |
