# Supplementary material for: Changes in isokinetic trunk muscle strength and endurance after two different restoration programs in people with chronic low back pain: A longitudinal retrospective study
Source: Heliyon. 2024 Jul 20;10(15):e34914. doi: 10.1016/j.heliyon.2024.e34914 (PMC11320202; doi:10.1016/j.heliyon.2024.e34914)
Supplement: Multimedia component 5 [file mmc5.pdf]

CERAPHP Centre

Comité d'éthique de la recherche AP-HP Centre

IRB registration : #00011928

Présidente : Marie-France MAMZER

Vice-présidente : Anne-Sophie JANNOT

Membres : Edouard Auclin, Christian BALLOUARD, Flore-Anne DE BAUDINIERE, Jean-Claude K. DUPONT, LISA FRIEDLANDER, Anne-Sophie JANNOT, Magalie LADOUCEUR, Ivan LERNER, Marie-France MAMZER, Tristan MIRAULT, Antoine NEURAZ, Nicolas PALLET, Tigran POGHOSYAN, Olivier PELLERIN, Brigitte SABATIER, Arthur TRON, Mar Vallès-Poch, David VEYER, , Aziz ZAANAN

On September 23, 2022, the Committee received a request from Dr. Alexandra Roren concerning a retrospective study entitled:
2022-10-12: Variation in spatiotemporal parameters of postural stability and gait in people with chronic low back pain after a supervised rehabilitation program (MarchStaLomb). Alexandra Roren

Paris, le 31/05/23

Réf. 2022-10-12

Le Comité a été saisi le 23 septembre 2022 d'une demande du Dr Alexandra Roren concernant une étude rétrospective intitulée :

2022-10-12 : Variation des paramètres spatio-temporels de la stabilité posturale et de la marche chez les personnes lombalgiques chroniques après programme de rééducation supervisé (MarchStaLomb). Alexandra Roren

Investigateur principal : Dr Alexandra ROREN
Service de Rééducation et Réadaptation de l'Appareil Locomoteur et des Pathologies du Rachis
Hôpital Cochin
27 rue du Faubourg Saint Jacques
75014 Paris

Après avoir confirmé que la recherche envisagée n'était pas une recherche impliquant la personne humaine au sens de la loi française n° 2012-300 du 5 mars 2012 relative aux recherches impliquant la personne humaine, et après avoir reçu des compléments d'information satisfaisant à la suite des remarques qu'il avait émises, le comité a rendu un **avis favorable le 10 février 2023**.

After confirming that the proposed research was not research involving the human beings within the meaning of French law no. 2012-300 of March 5, 2012 on research involving the human person, and having received satisfactory additional satisfactory additional information in response to the remarks it had made, the committee issued a favorable opinion on February 10, 2023.

Pr Marie-France MAMZER
Présidente du CERAPHP Centre
