# Supplementary material for: Changes in isokinetic trunk muscle strength and endurance after two different restoration programs in people with chronic low back pain: A longitudinal retrospective study
Source: Heliyon. 2024 Jul 20;10(15):e34914. doi: 10.1016/j.heliyon.2024.e34914 (PMC11320202; doi:10.1016/j.heliyon.2024.e34914)
Supplement: Multimedia component 6 [file mmc6.doc]

**AUTHOR CHECKLIST *Authors of all papers reporting clinical research should submit this checklist together with their manuscript and the Reporting Guideline Checklist found on the EQUATOR site (***[***http://www.equator-network.org/***](http://www.equator-network.org/)***).***

This checklist identifies recognised guidelines for scientific reporting, which authors should use to prepare their manuscript *(required for systematic reviews and original research)*

| ***Standards of reporting*** | The editors require that manuscripts adhere to recognised reporting guidelines relevant to the research design used. These identify matters that should be addressed in your paper. Please indicate which guidelines you have referred to.  These are not quality assessment frameworks and your study need not meet all the criteria implied in the reporting guideline to be worthy of publication in the MATH. The checklists do identify essential matters that should be considered and reported upon. For example, a controlled trial may or may not be blinded but it is important that the paper identifies whether or not participants, clinicians and outcome assessors were aware of treatment assignments.  **You are also required to submit a checklist from the appropriate reporting guideline (available on the EQUATOR website (<http://www.equator-network.org/>) together with your paper as a guide to the editors.  *Reporting guidelines endorsed by MATH are listed below:* | **Guideline referred to** | **Checklist submitted[[1]](#footnote-2)**** |
| --- | --- | --- | --- |
| Randomised (and quasi-randomised) controlled trial | CONSORT – Consolidated Standards of Reporting Trials  <http://www.equator-network.org/reporting-guidelines/consort/> |  |  |
| Study of Diagnostic accuracy / assessment scale | STARD Standards for the Reporting of Diagnostic Accuracy studies  <http://www.equator-network.org/reporting-guidelines/stard/> |  |  |
| Systematic Review of Controlled Trials | PRISMA - Preferred Reporting Items for Systematic Reviews and Meta-Analyses  <http://www.equator-network.org/reporting-guidelines/prisma/> |  |  |
| Observational cohort, case control and cross sectional studies | STROBE **St**rengthening the **R**eporting of **Ob**servational Studies in **E**pidemiology  <http://www.equator-network.org/reporting-guidelines/strobe/> | X | X |
| Case Reports | CARE - Case Reports - <http://www.care-statement.org/downloads/CAREchecklist-English.pdf> |  |  |
| Statistical reporting | SAMPL - guidelines for statistical reporting – *no checklist exists currently but authors are encouraged to view the guidelines on the EQUATOR website* [*http://www.equator-network.org/reporting-guidelines/sampl/*](http://www.equator-network.org/reporting-guidelines/sampl/) |  |  |
|  | *Qualitative researchers might wish to consult the guideline listed below* |  |  |
| Qualitative studies | COREQ: Consolidated criteria for reporting qualitative research (<http://www.equator-network.org/reporting-guidelines/coreq/>) |  |  |
| Other (please give source) | In addition we provide the template for intervention description and replication (TIDieR) checklist and guide. | X | X |
| Not applicable (please elaborate) |  |  |  |

1. ^*^ [↑](#footnote-ref-2)
